# Opinions Urgently Needed

**Published:** 1921-01-22

**Authors:** 


### The Hospital

The Workers' Newspaper of Administrative Medicine and Institutional Life. Administration. National Insurance and Health.

No. 1807, Vol. LXIX. SATURDAY, JANUARY 22, 1921. PRICE TWOPENCE

### OPINIONS URGENTLY NEEDED.

It may be something of an extremist statement to claim that the present policy of the Ministry of Health has but one end—namely, a complete State-salaried Medical Service and the extinction of our present system of medicine. Whatever justification there may be superficially for some of the jeremiads of the medical correspondent of *The Times*, on the main point we must disagree with him—that is, if he really implies that Dr. Addison is out, by hook or by crook, to bring the medical profession entirely under State control. The situation, as we see it, approximates more closely to a reasonable Governmental desire to bring a greater range of the population under a panel—or, rather, an improved panel—system. For the health of the nation generally it would be better, officialdom appears to hold, if poorer-class medical practice were entirely systematised on the insurance-practice principle. Quite possibly it would.

But before attacking this project with every available journalistic weapon the country must take careful consideration of the question from all the rather sharp angles it presents. In the first place, the Ministry may tend to further the State scheme beyond the most advisable limit. After all, most of us incline to consider as the best our own solution of a problem, particularly if detailed evidence and expressed views are not readily forthcoming from other parties involved in the difficulty. Secondly, among our massed population, and inevitably Great Britain is becoming annually more crowded, there is little doubt that a systematised Medical Service, technically first-class, but also having the authority of the Government behind it, is probably the best, and, indeed, the only, way of dealing with the more serious health problems ahead. And, thirdly, the panel system, whatever degree of futility we ascribe to its past career, as it stands to-day is admittedly not equal to the task of dealing with these problems according to the best ideals.

From these facts, then, the direct inference seems to be that up to a certain grade in our population, from the financial status aspect, we need an improved Medical Service, and obviously this service must operate under the ægis of the Ministry. Above a certain financial status the people can and should obtain medical care and advice on the same lines and in the same sphere as they have always done. The great question for the people to consider is, Where shall the dividing line be drawn? The corresponding problem before the medical profession calls for decision between a part-time service, such as the panels of to-day represent, and a full-time, properly remunerated service in which a number of medical men are absorbed, the remainder continuing independent practice among the appropriate classes of patients. The situation at the moment is unsatisfactory, chiefly because neither of these points have been openly and fairly discussed.

The reasons why representative medical opinion cannot be taken have already been explained in these pages, and the somewhat conflicting attitudes of the Federation and of the B.M.A. briefly outlined, but at the same time it was insisted that union of these two would not hinder, but rather aid, the latter in fighting whatever battles, and in standing firmly by whatever principles, the "family" doctor considers necessary. In any case the B.M.A. should undoubtedly take up a very much more defined attitude on these matters, and act with indications more obvious to the average doctor that its first and only aim is to bring genuinely representative medical opinion to Dr. Addison's hearing. If these conditions could be assured, then, as *The Times* says, it (the B.M.A.) "would not long wait for the recognition which an exasperated nation is ready to give it."

But mention of this lay support brings us back again to the question of the "dividing line" between spheres of State-controlled and independent medical practice. The Press has for many months past given publicity to statements, emanating chiefly from "medical correspondents," upon freedom in choice of attendants and a thousand allied questions. These articles inevitably exert their public influence. But they go no distance at all towards ascertaining and making known the real attitude of the layman.

May we urge our contemporaries to give equal prominence to representative views from the people themselves? We do not doubt that the public would wish their doctors to be free and independent, in the sense that every Briton is supposed to be free. But catch-phrases are of little use at the present juncture, and we should be more than glad to see some considered lay opinions as to the most desirable form of Government health control.

In these pages we are always glad to give space to discussions, particularly politically unbiased discussions, of the many problems which concern the general hygienic welfare of the people. The public has received much gratuitous advice in the last few months on its medical needs and the health ideals it should cherish. Is it not time that its own opinions were more fully expressed? The moment is undoubtedly opportune.

